# Supplementary material for: Are carbon emissions trading and green financial instruments synergistic? -Comprehensive quantitative research based on content analysis
Source: PLoS One. 2024 Mar 7;19(3):e0298601. doi: 10.1371/journal.pone.0298601 (PMC10919601; doi:10.1371/journal.pone.0298601)
Supplement: S1 Data — (ZIP) [file pone.0298601.s001.zip › Data/地级市Z_F工作报告.zip!/║■▒▒/╗╞╕╘╩╨/└┤╫╘╣1⁄2╓┌║┼í╛┬φ┐╦╩2╛▌═°í┐_2014.docx]

各位代表：

　　现在，我代表市人民政府向大会报告工作，请予审议，并请市政协各位委员和其他列席人员提出意见。

　　一、2013年工作回顾

　　过去的一年，面对复杂多变的宏观环境、经济下行的巨大压力、历史罕见的高温干旱，在市委的正确领导下，全市上下迎难而上，砥砺奋进，扎实推进“双强双兴”，深入开展“三大行动”，经济社会发展总体平稳向好，主要经济指标保持两位数增长。

　　——经济发展有新成就。全年地区生产总值1332.6亿元，增长10.5%；规模以上工业增加值413.7亿元，增长13.8%；全社会固定资产投资1416亿元，增长28.4%；社会消费品零售总额626.4亿元，增长13.2%；地方公共财政预算收入79.98亿元，增长27.1%；城镇居民人均可支配收入18432元，增长9.94%；农民人均纯收入6966元，增长13.41%。居民消费价格涨幅3%，城镇登记失业率4%，人口自然增长率6‰，节能减排完成省定目标。

　　——结构调整有新进展。农业经济稳步发展。粮食生产实现“十连增”，畜禽、水产、蔬菜等主要农产品产量快速增长。启动实施现代农业“个十百千”工程，新增市级以上龙头企业55家，其中国家级1家，全市农产品加工业产值增长23%。农村土地流转面积327万亩，新增农民专业合作社1079家、家庭农场85家。农机综合作业水平达到62.3%，气象等防灾减灾能力提升。新增地标产品13个，组建了全国首家地标产品集散中心。工业经济加快发展。建成各类开发区、工业园区55个，入园企业3000多家。五大主导产业总产值首次突破千亿元，增长20.6%。蕲春李时珍医药化工等7个产业集群成为全省重点成长型产业集群。规模以上工业企业达到1134家，新增252家；亿元企业达到445家，新增107家。13家企业被评为省级“两化融合”示范企业，6家企业列入省制造业信息化示范企业。现代服务业、高新技术产业、外向型经济快速发展。编制了《黄冈市文化产业发展规划纲要》，总投资200亿元的恒天（黄冈）文化创意产业城等重大项目成功落户。罗田薄刀峰、黄梅四祖寺通过4A级景区初评，麻城成功创建全省旅游强市，我市首次荣获全省旅游发展先进市。全年接待游客1500万人次，实现旅游收入79.5亿元，分别增长23%和22%。启动编制《黄冈城区商业网点规划》，新建标准化农贸市场17个。阿里巴巴落户武穴、红安，淘宝网黄冈馆、电子商务孵化器启动。高新技术企业新增31家，总数75家，高新技术产业增加值增长18.9%，占地区生产总值比重提高到7.27%。外贸出口45133万美元, 实际利用外资6749万美元，分别增长29.4%和 89.2%。

　　——“三大行动”有新成效。争取项目扩投资。“双百”项目开工160个，完成投资412.5亿元，135个谋划项目前期工作取得重大进展。交通完成投资179亿元，再创历史新高。农田水利完成投资24.8亿元。城际铁路、黄冈长江大桥、黄鄂高速即将通车，麻阳和麻竹高速、长河流域治理、水土流失综合治理等重大项目进展顺利。大力招商引投资。新引进重点项目482个，到位资金496亿元。成功举办农业、文化旅游产业招商推介会，签约项目76个，协议投资710亿元。昆仑能源黄冈LNG项目即将投产，投资150亿元的晨鸣林浆纸一体化项目正式落地。服务企业增投入。政银企对接落实信贷投放378.5亿元，中小企业融资平台新增担保额55.4亿元，大别山产业发展基金投放12.7亿元，培育上市后备企业60家，11家企业在场外交易市场成功挂牌（托管），资本市场直接融资8.8亿元。企业用地、用电、用工等要素保障能力增强。

　　——城乡建设有新面貌。城镇化率提高1.5个百分点。市区建设不断加强。城东新区当年完成投资35.7亿元，核心区基础设施建设全面推进。遗爱湖公园建成景区9个。城区新改扩建市政项目32个。实施了黄州商城改造，启动了“双改”工程。“城管会战”成效显著。县市城区建设加快。以“两区互动”为重点，红安南部新城等县市城区扩容提质成效明显。小城镇发展加速。编制完成《鄂东沿江城镇带暨临江四城发展规划》。小池开放开发省级战略成效显著，浠水滨江新区加快发展。新农村建设扎实推进。完成347个村农村环境连片整治示范，100个村进入全省“千村整治计划”，54个村被评为全省新农村建设示范村。启动武穴城乡一体化试点。英山茶叶景观带被评为全国最美田园。生态文明建设力度加大。启动“绿满黄冈”行动，推进中心城区水域保护，建立PM2.5灰霾监测系统，全年空气质量优良天数300天。

　　——市校院所合作有新突破。着眼创新驱动调结构转方式，以聚集人才、科技创新为主旨，以央校、央院、央所、央企为依托，以“一园八基地”为载体，启动实施“黄冈·高等院校科研院所合作计划”。建立合作机制。武大、华科、中科院武汉分院、武汉邮科院等20所重点高校、科研院所与我市签订战略合作协议，56名主要领导、专家教授成为市政府咨询委员。加强人才引进。出台引进人才优惠政策，在武汉高校开展18场宣传推介活动，吸引国内72所高校2万多名学生报名，已开展三批实习实训。推动创新创业。“创天下”大学生创新创业俱乐部成立，并得到团中央、省直有关部门授牌。市政府成立创业引导基金，“湖北珞珈青年创业基金投资中心”落户黄冈，并联合组建了创业投资基金。培育创新主体。科技孵化器建成投入使用。新组建水产院士工作站、纺织行业创新联盟等国家、省级科研延伸平台12个。争取项目投资。上市公司烽火集团投资的烽火科技（黄冈）产业园正式落户。

　　——深化改革有新成果。大别山试验区实现市域全覆盖，国家《大别山革命老区振兴发展规划》即将出台。《黄冈大别山试验区生态补偿政策研究报告》通过环保部评审。6县（市）扶贫开发纳入国家规划。成功列入全国“智慧城市”试点，成为全国首批资源综合利用“双百工程”示范基地。组织实施了黄州再生资源综合利用示范区、龙感湖新能源与农业循环经济示范园、团风东坡粮油集团土地流转机制创新等专项改革试验。融资体制改革取得突破，城投二期债券获准发行，亚行贷款列入国家规划。行政审批制度、食品药品监管体制、医疗卫生体制、“营改增”等改革顺利推进。理顺白莲河水库管理体制进展顺利。

　　——社会事业有新进步。坚持民生优先。全年财政民生支出245.2亿元，占公共财政预算支出81.5%。新增五类市场主体5.5万户，新增城镇就业6.9万人，转移农村劳动力就业6.5万人。9.8万贫困人口实现脱贫。五项社会保险参保总人数281.5万人。建立城乡居民大病医疗保险，启动实施事业单位工伤保险，失业保险实现市级统筹。新农合参合率99.6%。全面落实高龄老人补贴政策。新改建农村福利院73所。保障性住房基本建成及竣工15635套，农村危房改造完工15810户。新建连村通组公路1850公里，解决53.5万农村居民饮水安全问题。坚持事业惠民。新改扩建乡镇公办幼儿园103所，建成义务教育标准化学校260所，4个县通过义务教育均衡发展评估验收，高中阶段毛入学率87%。高等教育、职业教育和民办教育加快发展。职教园区主体完工。成功举办第八届湖北省黄梅戏艺术节，承办挺进大别山漂流赛、热气球挑战赛、汽车场地越野锦标赛等国家级赛事。舞台剧《亮剑——大别山》在北京首演，《青春派》《全城高考》双获中国电影“华表奖”。荣获“中华诗词之市”、“中国书法城”称号。黄梅戏大剧院、体育中心建设顺利推进。基层医疗卫生服务体系建设不断加强，低生育水平保持稳定。基层自治组织、社会共治机制、源头治理体系、信访维稳制度建设进一步加强。深入开展“百日千人”安全生产大检查，安全生产、农产品质量安全、食品药品安全监管不断加强，“六大食品安全”工程、“放心早餐”工程深入推进。“六五”普法、平安黄冈、法治黄冈建设扎实推进。政府“十件实事”全面完成。

　　——政府自身建设有新变化。认真贯彻落实中央八项规定、省委六条意见、市委“八简两风”要求，坚决反对“四风”，启动百姓问政，强化治庸问责，政府会议、文件减少，“三公”经费支出下降。坚持依法行政，完善工作规则。自觉接受人大法律监督、工作监督和政协民主监督，办理市人大代表建议121件，办理市政协提案292件。建立目标考核、责任倒逼、督查问责等机制，狠抓工作落实。加强政务公开、财政监督、审计监督、行政监察等工作，规范权力运行。认真落实党风廉政建设责任制，加强政府投资项目和财政资金监管，规范公共资源配置交易，严肃查处违法违纪案件，促进廉洁政府建设。

　　与此同时，国防动员、民兵预备役、双拥工作不断加强，经济普查、志书编修、档案管理、外事侨务、对台、妇女儿童、人民防空、国有资产管理、民族宗教、防震减灾、对口援建等各项工作取得新成绩，中直、省直驻黄单位贡献突出。

　　各位代表！过去一年，成绩来之不易。一年来，我们坚持“竞进提质、效速兼取”不动摇，千方百计稳增长，扭转了经济下行态势；坚持“双强双兴”、“三大行动”不动摇，千方百计促发展，经济实力不断增强；坚持聚集人才、科技创新不动摇，千方百计促转型，市校院所合作不断深入；坚持先行先试、敢闯敢试不动摇，千方百计抓改革，发展活力不断增强；坚持以人为本、民生优先不动摇，千方百计惠民生，群众生活不断改善。这是市委带领全市人民、调动各方力量团结拼搏的结果。在此，我谨代表市人民政府，向全市人民、各位人大代表、政协委员，向各民主党派、工商联、无党派人士、人民团体、离退休老同志、驻黄部队和武警官兵，向关心支持黄冈发展的社会各界人士，表示崇高敬意和衷心感谢！

　　回顾过去一年，在我国经济处于增长速度换挡期、结构调整阵痛期和前期刺激政策消化期、增长持续走低的大背景下，我市主要经济指标仍然全面完成省定目标，增速高于全国、全省平均水平，但地区生产总值、规模以上工业增加值、社会消费品零售总额、城镇居民人均可支配收入等4项指标增速未能实现市四届人大二次会议确定的预期目标。我们更清醒地看到，全市经济社会发展中仍存在不少矛盾和困难，政府工作还有一些不足之处。主要表现在：经济总量不大，产业结构不优，经济基础不牢，稳增长压力较大；科技人才缺乏、创新能力不强，转型发展任务艰巨；资源要素约束趋紧，生态环境压力加大；改善民生任务繁重，城镇化水平滞后，公共服务水平不高；行政服务效能有待提高，发展环境有待优化。对此，我们将采取更加务实有效措施，努力加以解决。

　　二、2014年工作安排

　　今年政府工作的总体要求是：深入贯彻落实党的十八届三中全会、中央经济工作会、省委十届四次全会和市委四届九次全会精神，坚持“竞进提质、升级增效”的总要求，牢固树立“市场决定取舍、绿色决定生死、民生决定目的”的理念，着力改革创新、激发市场活力，着力“双强双兴”、开展“三大行动”，着力创新驱动、推动转型升级，着力绿色发展、建设美丽黄冈，着力改善民生、加快事业发展，努力实现经济持续健康发展、社会和谐稳定。

　　主要预期目标是：地区生产总值增长10％以上；规模以上工业增加值增长13%；固定资产投资增长23%；社会消费品零售总额增长13%；外贸出口增长10%，实际利用外资增长15%；公共财政预算收入增长16%；城镇居民人均可支配收入增长10%；农民人均纯收入增长11%；居民消费价格涨幅控制在4%以内；城镇登记失业率控制在4.5%以内；人口自然增长率控制在6.5‰以内；完成省定节能减排目标。

　　实现上述目标，挑战与机遇并存。当前，经济形势复杂多变，区域竞争日趋激烈，稳增长、调结构、促转型任务艰巨，我们将面临更多严峻挑战。但更要看到，党的十八届三中全会发出了全面深化改革的动员令，省委十届三次全会吹响了“建成支点、走在前列”的冲锋号，长江经济带开放开发上升为国家战略，国家大别山革命老区振兴发展规划即将实施，必将带来重大发展机遇；市委“双强双兴”、“三大行动”决策部署深入人心，一批功能性重大基础设施、支撑性产业项目将陆续开工、相继建成，必将提振发展信心、增强发展支撑。面对新的发展形势，我们必须清醒认识面临的挑战，切实增强机遇意识、进取意识、责任意识，坚持底线思维，以更大的智慧和勇气攻改革之坚、克转型之难，牢牢把握改革创新、转型发展主动权，努力打造“黄冈经济升级版”。

　　（一）坚持科学发展，推进“双强双兴”，加强“三大行动”

　　坚持一张蓝图绘到底，把“双强双兴”作为推动黄冈科学发展跨越发展的根本路径和战略抉择，凝神聚力，持之以恒，继续推进 “双强双兴”，深入开展“三大行动”。

　　强力发展工业。推动工业增量提质。打造大园区。坚持工业向园区集中、园区向城镇聚集，重点建设11个经济开发区、26个规范化工业园区、47个初具规模的乡镇工业集中区。支持黄冈经济开发区创建国家级开发区、龙感湖工业园升级省级市属园区、县（市、区）开发区创建省级高新区。发展大产业。改造提升传统产业，实施五大主导产业、七大重点产业集群三年行动计划，建设一批重大技改项目、示范工程，培育一批“两化融合”试点企业、示范项目，推动工业制造向智能化、网络化、数字化转变。加快培育新兴产业，大力培育节能环保、新型材料、电子信息、生物工程等产业，积极发展能源产业。建设大项目。加快138个重点工业项目建设，确保新增规模以上工业企业100家以上。重点加强32个投资10亿元以上的重大产业支撑项目建设。发展大企业。对年产值3亿元以上的企业、3年内有望过5亿元的成长型企业、固定资产投资5亿元以上在建企业，建立企业目录，实行“点对点”扶持。培育大品牌。实施质量兴市、品牌强市战略，争创精品名牌、驰名商标、著名商标和知名商标。

　　发展现代农业。大力实施现代农业“个十百千”工程。提高农业综合生产能力。落实惠农强农政策，推进农业政策性保险，实施土地综合整治，加强农田水利、农业信息化、农技推广和防灾减灾体系建设，提高粮食安全保障能力。提高现代农业发展能力。加快土地流转，鼓励种养大户注册登记家庭农场，开展农民专业股份合作社试点，鼓励工商资本进入种养业，创建现代农业示范园、农业标准化示范区、绿色食品示范区、旅游观光农业示范区。提高农产品加工转化能力。围绕特色农业、地标产品，引进和培植一批领军型龙头加工企业，打造百亿产业链条。加大农业结构调整力度。加强“菜篮子”工程建设，加快畜牧、水产养殖业发展。加大扶贫开发力度。启动实施148个村整村推进，解决8万低收入人口脱贫问题。加大新农村暨城乡一体化建设力度。开展新一轮“三万”活动，推进城乡公共基础设施一体化、基本公共服务均等化，加强乡村公路建设，加快实施农村“安饮工程”，推动农超对接、城乡商业连锁经营、农村标准化金融服务联系点建设。

　　发展文旅产业。开展“智慧旅游年”主题活动。以“智慧旅游”、“一卡通”整合旅游服务，以文化、旅游发展规划整合旅游资源，以大别山水、人文黄冈核心品牌整合市场营销，做大做强大别山旅游开发公司，推动全市文化旅游一体化融合、差异化发展、组合式营销。加快恒天（黄冈）文化创意产业城等文化产业项目建设步伐，支持各地特色文化产业园区、旅游景区建设。开展A级景区、旅游强县、旅游名镇、旅游名街、旅游名村创建活动，启动重点景区5A级创建，积极开展旅游标准化试点。加强与十大旅游客源地城市、全国百强旅行社对接。力争全年接待游客人次、旅游收入分别增长15%和18%以上。

　　统筹城乡发展。坚持以人为本、优化布局、生态文明、传承文化，走集约、绿色、低碳、智能的新型城镇化道路。高昂市区龙头，加快市区发展。坚持产业兴城，加速产城融合，加强工业园区、服务业聚集区规划和建设，加快市区一体开发重大工业项目建设，中国食品博览交易中心等区域性总部建设，增强市区产业聚集功能。建设水岸新城，理顺城东新区管理体制，力争核心区道路全部贯通、“九通一平”基础设施基本完成，道路绿化亮化、三台河两岸绿化全面启动，学校、医院、综合馆等公共服务设施建设同步推进，提高市区人口承载能力。打造文化名城，完成黄冈历史文化名城保护规划编制，将文化元素融入城市规划、设计、建设之中。建设生态之城，编制城区水网综合治理规划，规划建设“环城生态示范带”，推进中心城区水域保护，加快遗爱湖公园建设，启动环白潭湖绿化生态工程建设。建设宜居之城，加强老城区市政设施改造，建立商品房开发配建保障性住房制度，启动6个以上片区“双改”，加强城乡客运公交化改造，实现城区至城际铁路站场公交无缝对接，深入推进新一轮“城管会战”和“四城联创”。打造智慧之城，大力推进三网融合、4G网络建设、信息资源整合，加快构建智能化基本公共服务体系，建设“智慧城市”。统筹推进县市城区、小城镇、新农村建设。坚持“形态、文态、生态、业态”有机统一，推进县市城区扩容提质工程，抓好38个重点镇、21个文化小镇、109个美丽乡村建设试点。

　　加强“三大行动”。推进项目建设提速增效。以目标倒逼进度，以时间倒逼工期，确保在建项目按期完工，力争全年“双百”项目完成投资500亿元以上。抢抓国家重大战略实施、区域发展布局、投资政策调整机遇，向上争取一批投资项目。加强黄冈武汉交通一体化、北部山区和沿江地区快速通道建设，谋划推进黄冈（蕲春）机场、武穴长江大桥、蕲河航道疏浚、武汉新港江北铁路黄冈段、麻竹高速延长线、蕲春至太湖高速、大别山电厂二期等项目前期工作，争取巴水、举水、浠水干流防洪工程立项。提高招商引资质量。完善招商引资服务、考核机制，力争全年到位资金突破600亿元。创新招商引资方式，坚持专业招商、产业链招商、以商招商等市场化招商模式，引进一批产业配套项目、上下游关联项目，推动产业链式发展。深化央企、名企合作，力争在引进国内外知名企业、上市公司上有新突破。规范招商引资优惠政策，提高项目准入门槛，强化投资强度、税收额度、建设期限、环境影响评价等刚性约束，落实违约项目劝退制度。加强企业要素保障。组建中小微企业服务平台，开展企业帮扶活动。加强政银企对接，建立金融机构支持重点企业激励机制。组建企业上市服务平台，做好后备企业股权融资等工作，积极推进企业上市工作。发挥融资担保机构作用，加强企业融资服务。建立职业技术培训、人力资源市场、企业用工需求对接平台，缓解企业用工难。加强重点企业用电、用地保障。

　　（二）加快转型发展，坚持创新驱动，加强生态文明建设

　　把创新驱动作为根本力量和关键举措，把绿色发展作为必然要求和应有担当，加快转型发展步伐。

　　坚持创新发展。把创新驱动贯穿到经济社会发展全过程。加快产业转型升级。深化科技体制改革，推动科技与经济紧密结合，以技术推广加快发展优质、高效、高附加值的现代农业，以技术改造全面提升食品、纺织、造船、建材、窑炉等传统产业，以产业引进加快培育信息技术、高端装备等新兴产业，以技术创新加快发展信息消费、电子商务等现代服务业。培育提升创新主体。积极催生创新型企业，提升现有企业创新能力，使企业成为技术创新的需求主体、投入主体、研发主体和应用主体。力争高新技术企业突破100家。实施人才强市战略。创新人才培养开发机制，营造宽松成长环境，以高层次科技人才、高水平创新创业团体、高素质管理人才、高技能实用人才为重点，统筹抓好各类人才队伍建设。

　　推进合作发展。以市校院所合作为平台，推进“一园八基地”建设。加快高校科研院所产业园建设。完成园区规划设计，加强基础设施建设。以烽火科技（黄冈）产业园建设为引领，培育信息产业集群。积极承接高校、科研院所产业转移，加强与中关村、东湖高新区的对接互动。努力把园区建成高新技术产业聚集区、省级创新驱动发展示范区。加强大学生实习实训基地建设。完善大学生实习实训推介、衔接、管理、服务机制，并进一步向省外高校拓展，扩大规模，提高质量，完成1万名大学生实习实训计划。以实习实训带动人才引进。加强就业创业基地建设。结合产业升级，开发更多适合高校毕业生就业岗位。完善“绿色通道”，落实激励政策，支持企事业单位招聘录用大学生、引进高层次科技领军人才。举办竞赛、论坛、辅导、培训等活动，将“创天下”创新创业俱乐部打造成知识、信息、资本、人才汇集平台。综合运用信贷扶持、“人才公寓”等政策措施，支持人才初创企业。加强国家和省级科研平台延伸基地建设。提高企业自主创新能力，结合企业提升发展需要，引进分平台30家。加强科研成果转化基地建设。建立产学研用协同创新机制，建立技术需求库，聘用项目信息员，加强高校院所科技成果跟踪、对接，推动科技成果在我市产业化。发挥政府创业引导基金作用，进一步引入各类创投基金，加快科技孵化器建设。力争光谷联合黄冈科技城落地建设。充分发挥人才智库基地作用，强化科学决策、转型发展的人才智力支撑。加强优质生源基地建设。新增10所高中为高校优质生源基地，让更多黄冈学子到著名高校学习深造。加强农产品供应基地建设。整合全市资源，构建直销武汉高校的农产品销售网络，建立一批农校对接基地。加强留学回国人员创业基地建设。增强人才政策开放度，吸引留学回国优秀人才到黄冈创业发展。

　　推动绿色发展。坚持生态立市，创建生态市（县、区）。加强生态文明制度建设。严守耕地红线，实行违法用地约谈问责制度。落实最严格的水资源管理制度。严格执行主要污染物总量控制、环境影响评价、建设项目“三同时”等环境保护制度，坚决落实生态环境保护责任追究、环境损害赔偿制度。开展生态文明宣传教育活动。强力推进节能减排。建立县（市、区）节能减排指标考核体系。严格执行主体功能区规划，严禁不符合区域环境政策的开发活动，严格涉重金属等环境敏感项目准入。推进能源资源节约和循环利用，重点抓好工业、交通、建筑、公共机构等领域节能降耗。加强企业节能减排技术改造，重点抓好印染、化工行业废水治理，火电、水泥企业脱硝脱硫设施建设。继续加强石材行业专项整治。加强农业面源污染防治，实现万头（只）以上畜禽养殖场污染治理全覆盖。推广城乡垃圾收集转运处置体系，构建城乡再生资源回收利用体系。加强生态环境保护。实施地绿工程，开展“绿满黄冈”行动，创建“绿色生态示范县（市、区）”和绿色乡村、企业、机关；推进长江防护林、退耕还林等生态工程建设；加强龙感湖等湿地公园和地质公园、自然保护区、森林公园建设，保护生物多样性。实施水碧工程，争创水生态文明试点市，强化河湖库长制度，落实城乡水体、水域和饮用水源地保护责任制，推行湖、库渔业生产人放天养。实施天蓝工程，开展大气污染防治，加强汽车尾气、餐饮业油烟等污染防治，实施城市公交车LNG单燃料改造，启动重污染天气应急预警系统。实施土净工程，推进农村环境综合整治“两清、两减、两治、两创”活动，建设优美洁净农村环境。

　　“绿水青山是最好的金山银山”。我们决不能以牺牲环境为代价换取一时的“富足”，决不能为了眼前经济利益而斩断子孙发展的“后路”。加强生态文明建设，是我们的政治责任和历史使命！

　　（三）全面深化改革，激发市场活力，增强内生动力

　　激发市场活力。正确处理政府与市场关系。坚决简政放权，做到“三个一律”：市场机制能有效调节的经济活动，一律取消审批；面向基层且由地方管理更为方便有效的经济社会事项，一律下放；社会组织能参与承担的事项，一律转移给社会组织。扩大经济发达镇经济社会管理权限。推进事业单位分类改革，推动公办事业单位与主管部门理顺关系和去行政化，政府事务性管理服务原则上都要引入竞争机制，通过合同、委托等方式向社会购买。

　　培育市场主体。积极发展各类资本交叉持股、相互融合的混合所有制经济，实现产权多元化。废除对非公有制经济的一切不合理规定，支持非公有制经济健康发展。降低市场准入门槛，简化企业创办程序，实行筹建登记“先照后证”、工商注册“零收费”、注册资本“零首付”，完善扶持创业的优惠政策，形成政府激励创业、社会支持创业、劳动者勇于创业的新机制，推动全民创业、回归创业、大学生创业，加快小微企业和民营经济发展，力争新增市场主体5万户以上。实施民营经济增量提质工程，激励“个转企”、“私转股”，做大做强民营企业。

　　激活发展要素。推进金融改革创新。启动实施“黄冈大别山金融工程”，构建覆盖各产业、各县（市、区）的多层次金融工程体系，推动各类金融和泛金融市场发展。加快发展地方银行，引进银行分行级机构1家以上，设立村镇银行2家以上。大力发展普惠金融。改革政府性公共资金存放管理办法。在防控政府债务风险基础上，做大做强城市投融资平台。实施信用培植工程。推进土地制度改革。积极开展城乡建设用地增减挂钩、低丘缓坡土地综合开发利用改革试点，探索建立指标交易制度。做好农村土地承包经营权确权登记颁证工作，建立农村“三资”监管、土地流转服务、产权交易平台，赋予承包经营权抵押、担保权能，稳妥推进农民住房财产权抵押、担保、转让，逐步把农村经营性建设用地纳入统一土地交易平台。

　　推进改革试验。积极承接和实施各类改革试验、试点示范。推动“两型”社会综改区体制机制创新。加快全国资源综合利用“双百工程”示范基地建设。深入推进服务业综合改革，支持省级服务业综合改革试点和示范园区建设，落实服务业要素同价政策，推进商品服务、商业模式创新。抢抓大别山试验区上升国家战略机遇，争取大别山生态补偿机制进入国家试点，努力建设绿色发展先行区。

　　提高开放水平。构建开放型经济体系。积极应对武冈城际铁路通车、黄冈武汉同城化带来的机遇与挑战，主动加强与武汉的大光谷、大汽车、大临空、大临港对接合作，把黄冈建成武汉产业转移的接地城市、农副产品供应的基地城市、物流发展的节点城市和旅游休闲目的地城市。抓住长江经济带开放开发、长江中游城市群建设等国家战略机遇，科学谋划长江（黄冈）岸线资源开发和武汉新港黄冈港区建设，大力推进沿江城镇群建设，加快小池开放开发省级战略实施，打造黄冈经济新的增长极。加大外贸主体培育力度，加强出口基地和通关体系建设。

　　（四）增进人民福祉，发展社会事业，创新社会治理

　　坚持民生优先，织好民生保障网。把促进就业作为经济发展优先目标，建立健全政府促进就业责任制度，全年新增城镇就业5万人以上，转移农村劳动力5万人以上。重点抓好高校毕业生、失地农民、困难人员就业工作。完善城镇居民收入增长促进机制和最低工资标准调整制度，推行企业工资集体协商制度，完善低收入群体物价补贴联动机制。落实征地拆迁补偿政策，建立企业拖欠农民工、职工工资“黑名单”制度。进一步扩大社会保障面，完善“五险一金”核定、征缴、管理体制，推进社保体系从制度全覆盖到人口全覆盖。完善被征地农民养老保险政策。实施医疗、工伤、生育保险市级统筹。整合城乡居民基本医疗保险制度，健全全民医保体系。发展社会福利和慈善事业，开展“社会救助管理创新年”活动。建立城乡社会养老服务体系，加快养老服务业发展。完善农村留守儿童、妇女、老年人服务体系，健全残疾人权益保障、困境儿童分类保障制度。加强和改进城乡低保、农村五保、困难群众生活救助制度。加强未成年人保护。加强保障性安居工程建设。

　　发展事业惠民，满足群众公共服务需求。办人民满意教育。深化教育综合改革，建设教育强市。坚决按要求落实教育投入政策，坚决按政策落实教师待遇。着力推进中小学校管理体制和教学改革，建立完善教师（校长）补充、交流、培训、考评机制。实施新一轮学前教育三年行动计划、义务教育标准化学校建设工程和农村教学点师资质量提升工程。加强职业教育基础能力建设，推动高等教育资源整合。积极发展特殊教育、继续教育、民办教育。加快振兴黄冈中学，擦亮黄冈教育品牌。坚持文化惠民。深化文化管理体制改革，激发全社会文化创造力。实施“乡村记忆”工程，加强文化遗产保护。创建国家公共服务文化示范区项目，促进文化产品、文化服务与群众文化需求有效对接。提高人民健康水平。深化医药卫生体制改革，扩大公立医院改革试点，完善基本药物制度，加强基层卫生机构和村医队伍建设，健全分级诊疗模式。坚持计划生育基本国策，综合治理出生人口性别比偏高问题。广泛开展全民健身活动。

　　维护民生安全，提高社会治理能力。建立隐患排查治理体系、安全预防控制体系，推行“智慧安监”，强化安全生产责任制，坚决遏制较大以上安全事故。用最严谨的标准、最严格的监管、最严厉的处罚、最严肃的问责，加强农产品和食品质量安全监管，确保老百姓“舌尖上的安全”。建立健全社会治理体制，创新社会治理方式，提高社会治理水平。建设覆盖城乡的信息化、网格化社会管理体系。做好村（居）民委员会换届工作。健全基层信访工作网络、网上受理信访制度，完善信访矛盾源头化解、依法治理机制。争创全国法治城市。深入推进平安黄冈建设，创新立体化社会治安防控体系，依法防范和惩治各类犯罪活动，维护社会和谐稳定。

　　推进军民融合深度发展，落实军地协调、需求对接、资源共享机制，深化国防教育和民兵预备役体制改革，加强征兵和双拥工作，巩固军政军民团结，努力为驻黄部队、武警和消防部队建设创造良好条件。

　　注重民生关切，继续坚持为民办实事。完成597个村卫生室“五化”标准建设。免费开展计划怀孕夫妇孕前优生健康检查、农村适龄妇女“两癌”（乳腺癌、宫颈癌）筛查。建成200所义务教育标准化学校、132所乡镇公办标准化幼儿园，推进市区学校布局调整和“四校一基地”建设。新开工建设及改造各类保障性住房3万套（户），完成保障性安居工程40万平方米。加快推进养老服务体系建设，全面开通12349居家养老信息服务平台，为万名以上居家老人提供便民服务，新建社区居家养老服务中心50个、农村老年人互助照料活动中心100个。免费为1000名贫困残疾人配发辅助器具，为300名0-6岁贫困残疾儿童实施抢救性康复，为2万名特困残疾人发放生活补助。新建连村通组公路1200公里。解决12万人饮水安全问题。为困难群众和农民工提供法律援助，办理援助案件1500件，其他法律援助事项15000件。完成150个村农民体育健身工程建设。创建市级农资经营示范店50家、村级综合服务社400家。

　　增进民生福祉、促进社会公平，是政府工作的出发点和落脚点。作为革命老区、贫困地区，我们更应该坚持经济发展促民富、事业发展惠民生、城乡建设增民享、扶危济困解民忧、维护稳定保民安，让改革发展成果更多、更公平地惠及全市人民！

　　（五）坚持群众路线，不断改进作风，加强政府自身建设

　　以服务群众为根本，建设服务型政府。深入开展群众路线教育实践活动，把群众的呼声、意愿、诉求作为施政理政的终极导向，面对面解决问题，实打实为民服务。坚持问题导向，从群众不满意地方改起，健全改进作风常态化机制，深入推进“四风”专项治理，继续开展百姓问政、行风热线等活动，下决心改会风、整文风、转作风，严肃查处不作为、慢作为、乱作为的人和事。

　　以执行落实为核心，建设效能型政府。推行服务承诺、首问负责、限时办结等制度。深化电子政务应用，加强电子监察，再造行政服务流程，实现“网上办事”便民化、行政审批规范化、政务服务高效化。完善政府绩效考核，加强政府绩效管理，强化治庸问责、约谈问责，提高政府执行力、落实力。

　　以解放思想为先导，建设创新型政府。坚持理念创新，冲破思想观念的障碍，跳出条条框框的限制，克服部门利益的掣肘，以自我革新的勇气和胸怀推动政府职能转变，只要有利于黄冈发展，有利于民生福祉，就大胆试、大胆闯，坚决破、坚决改。

　　以依法行政为重点，建设法治政府。坚持依法决策，完善重大行政决策听证、合法性审查和风险评估等制度。坚持重大事项向人大报告、与政协协商制度，虚心接受舆论监督、群众监督。以法治理念做好人大议案、建议和政协提案办理工作，提高办理工作效率和质量。完善行政执法体制，强化食品药品、安全生产、环境保护、劳动保障等重点领域基层执法力量。规范行政执法行为，加强执法监督，严格执行执法责任制、执法违法责任追究制。

　　以强化制度为保障，建设廉洁政府。认真落实党风廉政建设责任制，严格执行各项纪律规定。贯彻厉行节约反对浪费条例，推进部门预算和“三公”经费预算公开，继续严控“三公”支出。加强行政监察和审计监督，完善公共资源配置、交易和管理，严肃查处重点领域违纪违规问题。全面落实部门权力清单制度，健全行政权力规范运行长效机制，把权力关进制度的笼子。加强政务、事务公开，推进决策、管理、服务、结果公开，让权力在阳光下运行，以公开促公正，以透明保廉洁。

　　各位代表！当前黄冈正处于科学发展、转型发展的关键时期，全市人民充满期待，我们重任在肩、使命光荣。我们将倍加珍惜时代赋予的机遇和平台，倍加珍惜全市人民的信任和支持，在市委的坚强领导下，坚持开拓进取，锐意改革创新，努力谱写“四个大别山”建设新篇章!
